# Influence of Non-lethal Doses of Natural Insecticides Spinetoram and Azadirachtin on *Helicoverpa punctigera* (Native Budworm, Lepidoptera: Noctuidae) Under Laboratory Conditions

**DOI:** 10.3389/fphys.2020.01089

**Published:** 2020-08-28

**Authors:** Anja Betz, Nigel R. Andrew

**Affiliations:** ^1^Insect Ecology Lab, Zoology, University of New England, Armidale, NSW, Australia; ^2^Institut für Insektenbiotechnologie, Justus-Liebig-Universität Giessen, Giessen, Germany

**Keywords:** *Helicoverpa punctigera*, native budworm, Spinetoram, Azadirachtin, natural insecticides, thermolimit respirometry

## Abstract

*Helicoverpa punctigera* (native budworm) is an important pest species in crops across Australia. From the third instar onward, this species causes severe damage to crop plants: therefore, caterpillars need to be managed at an early stage of their development. In our experiment, we raised *H. punctigera* on an artificial diet, which included different concentrations of the natural insecticides Spinetoram and Azadirachtin. The survival of the larvae, growth and body mass gain was recorded over 17 days. Only caterpillars raised on lowest toxin concentrations survived and molted successfully to the fifth instar, but had slower growth and body mass gain compared to the insecticide-free control group. Caterpillars fed on higher toxin concentrations never molted to the next instar or died in the first few days. To test how the toxins influence physiological conditions including metabolic rate and water loss, surviving fifth instar larvae were exposed to thermolimit respirometry: starting at 25°C following a constant increasing temperature ramping rate of 0.25°C^–1^, until reaching the critical thermal maxima (CT_*max*_). Caterpillars raised on a non-lethal dose of insecticides had higher metabolic rates and lost more water compared to the control group. Insects that have seem to consume more energy per mg tissue and have a higher water loss at high temperatures. Non-lethal concentrations of insecticides on pest insects physiology may reduce their impact on crops and may enable more targetted insecticide application.

## Introduction

Arthropod damage is estimated to be a loss of between 18 and 26% of annual crop production worldwide (Culliney, 2014). The Food and Agriculture Organization of the United Nations (FAOSTAT) estimates a total pesticide use of 50921.6 tons per year for Australia (2016), of which 22% are insecticides (FAOSTAT, 2016). In addition, the use of synthetic pesticides has become increasingly challenging since over 500 species of arthropod pests have evolved resistance to one or more insecticides or the pesticides have been removed from large-scale usage due to the hazards that they pose to the environment and non-target species (Chandler et al., 2011). Biopesticides, supposed to be more environmentally friendly than standard pesticides, are classified according to their active ingredient including microorganisms, biochemicals and semiochemicals (Chandler et al., 2011).

Currently, there are various biopesticides, including the bacterium *Bacillus thuringiensis* (BT) toxins and Spinosad (Spinosyns A and D). Here, we are interested in Spinosyn-derived insecticides and Azadirachtin. Spinosyns are derived products from the actinomycete *Saccharopolyspora spinosa* and are mainly used to control Lepidoptera and Diptera species (Shimokawatoko et al., 2012). Due to increasing insect resistance, synthetic modifications on Spinosad has resulted in a recently formulated insecticide – Spinetoram – that is also considered a natural insecticide (Visnupriya and Muthukrishnan, 2016). Spinetoram is a synthetic modification of the Spinosyns J and L and was brought onto the market in 2007 (Dripps et al., 2008). Spinosyns mode of action distinguishes them from other insecticides, by acting on a different position on the nicotine receptor compared to the nicotinoids insecticides (Dripps et al., 2008). The other insecticide used in this trial was Azadirachtin, which is the active ingredient of Neem oil: which is extracted from the Indian Neem tree *(Azadirachta indica*), that is native in the Indian subcontinent (Isman et al., 1990). The highest natural occurring yield of Azadirachtin found in the neem kernels is approximately 10 g/kg seed (Mordue and Blackwell, 1993). Azadirachtin is an ecdysteroid: these are hormones that can inhibit larval development by disturbing the molting process. It has a deterrent, anti-ovipositional anti-fecundity, antifeedant, growth-disrupting and fitness-reducing influence on insects (Schmutterer, 1990). Since Azadirachtin is a naturally occurring plant extract, it is used against different insect species (Chandler et al., 2011), but only one study has been conducted with *Helicoverpa armigera* and none on *Helicoverpa punctigera* (Ma et al., 2000).

To determine how a non-lethal dose of insecticides can influence the physiology of an insect, we chose a common Australian Lepidoptera pest species *H. punctigera*. This species is native to Australia and uncommon in other countries (Gregg et al., 2018). Adults of the *H. punctigera* can migrate long distances, adapt to different environments and have a wide range of host plants. In Australia, they have become a critical pest species in Queensland, Western Australia and South Australia (Gregg et al., 2018). Under warm temperature conditions of 25°C, the larvae only need about 2–3 weeks to become an adult (Gregg et al., 2018). Adults feed on nectar, living for approximately 10 days, in which females can lay up to 1000 eggs. Eggs are laid on leaves, flower buds, stems, and developing fruits of their host plant (Zalucki et al., 1986). The first instar hatches under dry and warm conditions within 3–4 days and starts feeding immediately on foliage before moving on to flower buds, flowers, pods, fruits and seeds, as they mature (Gregg et al., 2018). The successful control of the larvae has to be done in the early developmental stages since from their third instar on they cause the most damage to the crop plant. Furthermore, they have become more abundant in the cotton-growing season due to their resistance of BT-toxins, their ability to migrate, and temperature tolerance (Gregg et al., 2018). In Africa, Australia and Europe the related species *H. armigera* is a common pest (Gregg et al., 2018). *H. armigera* has already exhibited resistance to all conventional insecticide classes such as organochlorine, organophosphate, carbamate and pyrethroid insecticides (Wang et al., 2009). Since *H. armigera* is closely related to *H. punctigera*, resistance could also become an issue.

To minimize water loss, particularly at high temperatures, insects have adapted their respiratory system, excretory system, behavior and cuticle (Williams and Bradley, 1998). An insect’s natural ability to control water loss may be undermined with additional exposure to insecticides. Therefore, it is vital to understand how a small dose of a natural insecticide influences the physiological conditions of crop pests without polluting the environment unnecessarily.

Metabolic rate and water balance are closely related aspects of insect physiology. Some insects can prevent water loss with discontinuous gas exchange (Williams and Bradley, 1998). Assessing insect metabolic rates (oxygen uptake or carbon dioxide release) gives information about the energy consumption of the organism (Harrison, 2009). The metabolic rate of insects is influenced by developmental stage, activity, body mass (Harrison, 2009), temperature (Lighton, 1989), insecticide exposure (Janssens et al., 2018) and likely many other factors. Experimentally, it is critical to find the right scale of temperature exposure for small insects (Terblanche et al., 2007). The slower the temperature rises, the higher is the risk of a heat shock, which might not have occurred under the animal’s natural exposure (Lutterschmidt and Hutchison, 1997). However, the faster the ramp is, the higher the animal’s body temperature may be, leading to an overestimation of their critical thermal maximum (CT_*max*_) (Lighton and Turner, 2004). For these reasons, we decided to use the protocol according to Lighton and Turner (2004), which sets the temperature and exposure time depends on the body mass and surface area of the insect.

Assuming that most insecticides are applied to control early larval stage damage, we fed *H. punctigera* caterpillars in early developmental stages with different concentrations of Spinetoram and Azadirachtin in an artificial diet. We assessed how the toxin exposure affects larval growth, daily body mass gain and survival when applied at an early stage. Furthermore, we tested, the effects of various non-lethal insecticide dose exposures on the physiological condition of the caterpillars (i.e., for caterpillars that survived toxin exposure). The physiological condition was assessed via water loss and metabolic rates at increasing temperatures until they reached the critical thermal maxima (CT_*max*_) using thermolimit respirometry (Lighton and Turner, 2004; Le Quéré et al., 2016). We predicted that feeding the caterpillars low insecticide concentration diets would disrupt the physiological conditions of the insect (survival, metabolic rate, water loss, CT_*max*_) and this disruption would become more pronounced under high-temperature exposure.

## Materials and Methods

### Husbandry

*Helicoverpa punctigera* eggs were collected from a breeding colony (Tamworth Agricultural Institute, New South Wales, Australia), sterilized with a 2% bleaching solution, and washed with distilled water. After washing, water was removed through a vacuum pump, and eggs were collected on filter paper, which was placed in the lid of an upside-down 500 ml plastic cup, thinly covered with artificial diet (see below). To absorb water condensation, a sheet of tissue paper was placed into the cup and changed daily. After 4 days, the first instars were separated into 50 ml plastic cups containing 10 g of artificial food (ingredients listed below). The experiments were conducted in a temperature-controlled room at 25 ± 2°C at a 12:12 LD photoperiod (300 Lux).

To produce 500 ml of artificial diet, 200 g of soybeans were soaked overnight and cooked for 10 min in a pressure cooker. Subsequently, 40 g wheat germ, 35 g brewer’s yeast, 2.2 g nipagin, 1.1 g sorbic acid, 9 ml 10% formaldehyde solution, 3.5 ml mold inhibitor, 5 g agar and 500 ml distilled water were added in a food processor. After the food mass had cooled to room temperature, 3.5 g of ascorbic acid was added.

### Experiment 1: Spinetoram Exposure

For the control group, 15 plastic cups were filled with 10 g of the prepared diet each. The treatment consisted of six concentrations of Spinetoram (water dispersible granule containing Spinetoram-J and-L, Dow AgroSciences Australia). These were mixed to the artificial diet to obtain concentrations of 0.01, 0.04, 0.08, 0.20, 0.40, and 0.60 mg Spinetoram per liter diet with 15 replications of each treatment. Larvae were transferred into the experimental cups 1 day after hatching.

### Experiment 2: Azadirachtin Exposure

For the Azadirachtin (Sigma-Aldrich, 2 × 5 mg) experiment, ten replicates in each concentration (10, 30, and 60 mg/L) and ten replicates for the control group were used. Azadirachtin was mixed with artificial diet to gain desired concentrations, the control group was fed with an artificial diet without Azadirachtin.

### Growth

Larvae were inspected for movement to determine death. Furthermore, throughout both experiments, the length of each larva was measured every 2–3 days under an imaging stereo–microscope (Leica MZ16 A, Leitz Wetzlar, Germany). Also, for the Azadirachtin experiment, the body mass of each larva was recorded with a microbalance (0.001 g; Mettler Toledo, XP2U, Switzerland) again every 2 to 3 days.

### Food Consumption

To determine the amount of food consumed by each larva throughout both experiments, first, the weight of the food in each container was measured on an analytical balance (0.0001 g; Mettler Toledo, XP404S, Switzerland). Second, the water content in the diet was determined by drying four aliquots of diet at 60°C over 48 h. Third, after the larvae had either died or had been removed at the end of the experiment, the remaining food was dried and weighed.

### Metabolic Rate and Thermal Limits

At the end of both experiments, the thermolimit respirometry (Lighton and Turner, 2004; Andrew et al., 2016) was assessed in the fifth instar of caterpillars in the exposure of lowest concentration of Spinetoram and Azadirachtin as well as their control groups. Metabolic rate, activity and water loss were measured over a 25–55°C temperature range in a water bath (Type R4, Grant Instruments, Cambridge, United Kingdom). The experimental protocol followed the procedure described by Andrew et al. (2016). In short, after placing the larva into the respiratory chamber (15 ml) the temperature was maintained at 25°C for 10 min before ramping the temperature by 0.25°C/min up to 55°C when the larva was removed from the chamber after 130 min. Larval activity in the respirometry chamber was recorded with an infrared activity detector (AD2-1201-08, Sable Systems International). Metabolic rate (*V̇*CO_2_) and water was continuously recorded in a flow–through system with a Li-7000 analyzer (Li-Cor, Lincoln, NE, United States). The outside air was scrubbed of H_2_O (Drierite) and CO_2_ (soda lime). The flow rate was maintained at 200 ml/min with a mass flow controller (Sierra, 840L-2-OV1-SV1-E_V1-S1) that was checked before each measurement against a mass flow meter (Universal Gas Flowmeter, ADM1000, Agilent Technologies). The temperature in the chamber was recorded with a T-thermocouple inserted into the respiratory chamber, and connected to a data–logger (TC-08, Pico Technology, United Kingdom). To compensate for analyzer drift, baseline recording was performed before and after each experimental run for 5 min, and data acquisition occurred at 1-second intervals. Mass-specific metabolic rate and mass-specific water loss values were extracted over the whole 130 min and at temperatures of 25°C, 30°C, 35°C, 40°C, and 45°C ± 1°C. To measure temperature stress in the insects, three different thermolimit stages were extracted for metabolic rate; Equilibration; Ramping; Pre-mortal plateau (Lighton and Turner, 2004; Stevens et al., 2010; Andrew et al., 2016). The stress phases were extracted with Expedata (Sable Systems International) categorized by breeding amplitude of the animal. Equilibration phase marks the stage in which the insects perform under little stress. In the ramping phase, the frequency of the CO_2_ amplitude is higher, and stress is increased. The pre-mortal stress phase is the period just before the insect reaches CT_*max*_ is when the insect is under extreme thermal stress.

### Data Analysis

Statistics were performed in *R* 3.3.2 (R Foundation). For the survival analysis individuals that had survived the trial were censused (i.e., time of death was not determined; Library *survival).* Differences in growth and metabolic rate for different temperatures were assessed using a mixed-effects model (Library *nlme*) with individuals coded as a random factor to account for the repeated measure design. Body mass and body length were log-transformed before testing. For models with more than two factors, only first-order interaction terms were considered. Model selection was based on AIC, as this selected the most parsimonious model over the total experiment. This approach was simplified to a Students *t*-test for food consumption, CT_*max*_, total mass-specific metabolic rate and total mass-specific water loss as a sufficient number of larvae had survived only from the lowest toxin concentration and control. Metabolic rate and water loss over the experimental run were integrated across all measurements after baseline correction. CT_*max*_ was characterized as an inflection point in for CO_2_ absolute difference sum (ADS) residuals (Lighton and Turner, 2004; Andrew et al., 2016). Data are presented as the mean ± SD.

## Results

### Experiment 1: Spinetoram Exposure

**FIGURE 1:**
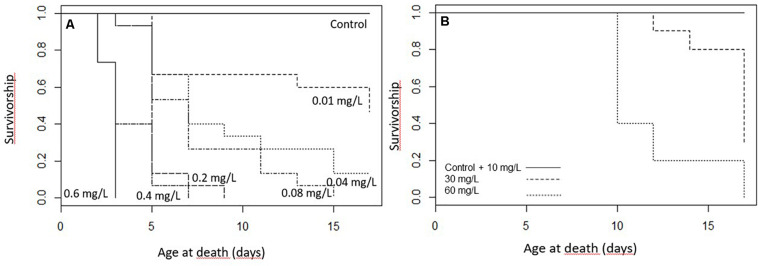
The survivorship of the caterpillars during the treatment **(A)** Spinetoram treated larvae (0.6 mg/L, 0.4 mg/L, 0.2 mg/L, 0.08 mg/L, 0.04 mg/L, and 0.01 mg/L) and the control group, **(B)** larvae treated with 10 mg/L, 30 mg/L, and 60 mg/L Azadirachtin and the control group.

The survival of the caterpillars decreased with increasing toxin concentration in the diet (*z*_101_ = −7.85; *p* < 0.001; Figure 1A). In the exposure of 0.6 mg/L Spinetoram, all larvae died within the first 2 days after the trial started and only one caterpillar reached the second instar. At the lower concentrations of 0.2 mg/L Spinetoram, five larvae died within the first 2 days of the trial, and only one molted to the second instar. After 4 days only one first-instar caterpillar was alive, which died 2 days after. At a concentration of 0.4 mg/L Spinetoram eight of fifteen larvae reached their second-instar after 2 days of treatment. On day 4, only 2 second-instar caterpillars remained, which died 2 and 4 days after.

In the Spinetoram concentration of 0.08 mg/L, four of fifteen larvae reached second-instar 2 days after the trial started, and two died. On day 4, only 4 second- and four first-instars remained. On day 6, one reached third-instar, while another four larvae died; leaving another 2 second- and one first-instar larva. After 8 days, one reached forth-instar, while others remained in their current instar. After 10 days only a forth- and second-instar remained; the second one died 3 days after and the forth 15 days after the trial started.

In the treatment of 0.04 mg/L Spinetoram, one larva died after 2 days and 30% molted to the second instar. After 4 days, 40% of the caterpillars died; and after 8 days, three of the six remaining caterpillars had reached third-instar. On day 10, one caterpillar had molted to fourth-instar, and another one died. On the following days, four more caterpillars died, and the two remaining caterpillars reached their forth-instar when the treatment ended.

In the lowest concentration of 0.01 mg Spinetoram per liter diet, five of fifteen larvae molted to second instar after 2 days. After 4 days, four larvae died; after 6 days, five larvae had molted to third-instar, and the other remaining six stayed in their second-instar. At day 10, all remaining caterpillars reached third-instar and the two of them the fourth-instar. At day 13, one more died and two more molted to fourth-instar. By day 15, three larvae had reached the final stage and the others except one molted to fourth-instar. By the end of the trail (17 days) another two died and two others reached the final stage.

As a comparison, none of the larvae in the control group died, 60% molted into second-instar after 2 days. After 4 days all caterpillars had reached second-instar and seven of them had molted to third-instar. After 6 days all caterpillars had successfully molted to third-instar and two of them to fourth-instar. At day 8 all caterpillars had reached at least their forth-instar and four of them had reached the final stage. After 13 days all caterpillars of the control group had reached there fifth and final instar.

**FIGURE 2:**
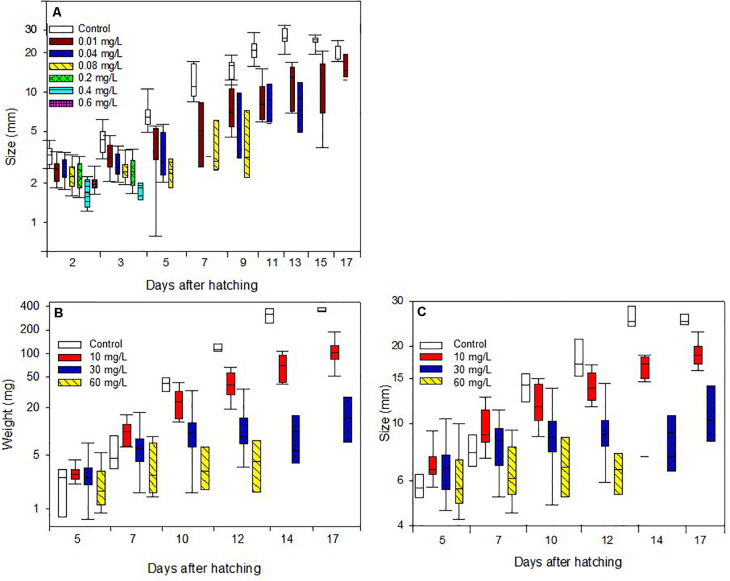
Body length increase of caterpillars **(A)** treated with Spinetoram, **(B)** body mass increase, and **(C)** body length increase of caterpillars treated with Azadirachtin.

Results showed that Spin**e**toram treatment depending on the concentration did not cause immediate death in the larvae, but inhibited caterpillars from maturing to the next stage compared to the control group. Furthermore, Spinetoram inhibited the growth of larvae (Figure 2A and Table 1A). the most parsimonious model included toxin concentration, time with an interaction term. As such, even the larvae fed on the lowest concentration attained approximately only half the length of the control individuals through most of the experiment.

**TABLE 1:**
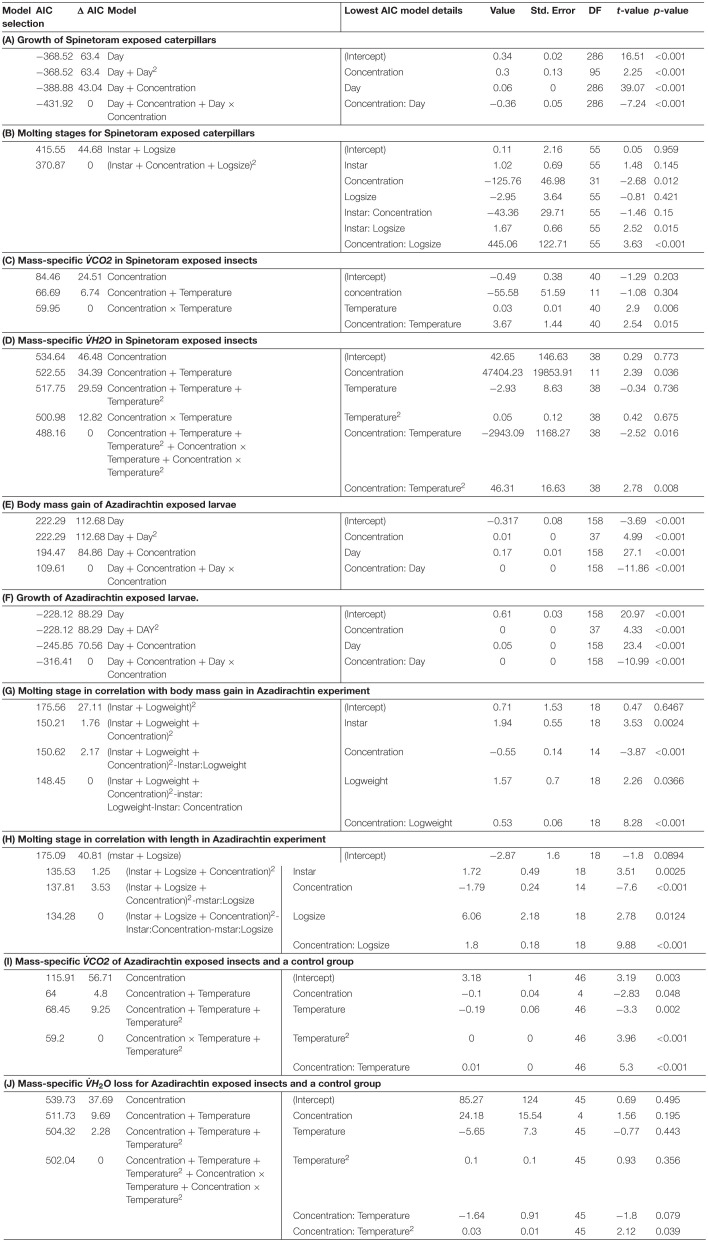
Model selection for (A) growth of Spinetoram exposed caterpillars; (B) moulting stages for Spinetoram exposed caterpillars; (C) mass-specific *VCO*_2_ in Spinetoram exposed insects; (D) mass-specific *V̇H*_2_*O* in Spinetoram exposed insects; (E) body mass gain of Azadirachtin exposed larvae; (F) growth of Azadirachtin exposed larvae; (G) molting stage in correlation with body mass gain in Azadirachtin experiment; (H) molting stage in correlation with length in Azadiractin experiment; (I) mass-specific VÌĞCO2 of Azadirachtin exposed insects and a control group; and (J) mass-specific *V̇H*_2_*O* loss for Azadirachtin exposed insects and a control group.

Increasing concentrations of Spinetoram delayed the timing of molting for all instar stages and head capsule size. The size of the larvae also affected the timing of molting (Table 1B). Larvae exposed to higher Spinetoram concentration molted later; or in the case of the very high concentrations did not molt at all.

Since only larvae of the control group and the lowest concentration sufficiently survived the trial, the dry mass consumed was compared in the two groups. Caterpillars fed with 0.01 mg/L Spinetoram had consumed significant less (*t*_23_ = −3.83; *p* < 0.001; 0.158 ± 0.07 g) than the control group (0.2624 ± 0.07 g).

### Metabolic Rate and Thermal Limit

**FIGURE 3:**
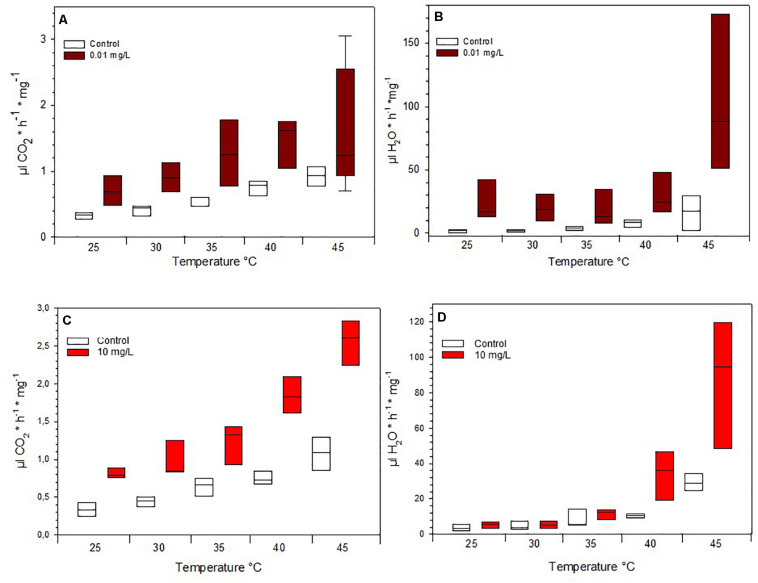
Mass-specific metabolic rate of **(A)** Spinetoram exposed insects and a control group at different temperatures and **(C)** of Azadirachtin exposed insects and a control group. Mass-specific water loss of **(B)** Spinetoram exposed insects and a control group at different temperatures and **(D)** in Azadirachtin exposed caterpillars and a control group.

**FIGURE 4:**
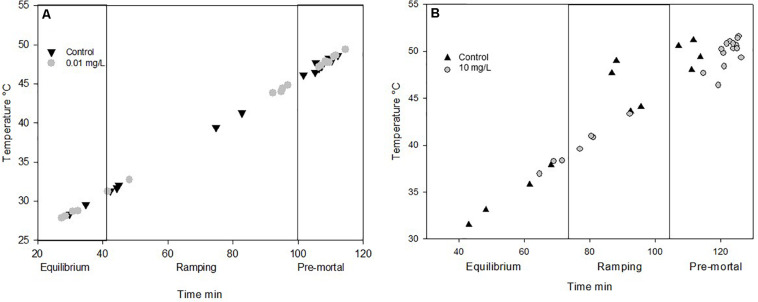
**(A)** Different stress phases in Insects during ramping phases of **(A)** Spinetoram experiment and **(B)** Azadirachtin experiment. Points mark the end of the phase of each individual. Equilibrium marks the no stress phase. In the ramping phase the insects starts to respire in a higher frequency with raising temperature. At the pre mortal phase the insect is about to die.

Due to mortality, only the lowest concentrations fed on 0.01 mg/L Spinetoram could be compared with the control group. The larvae raised on Spinetoram were significantly smaller, metabolic rate as well as water loss per mg body mass, both were significantly higher in the Spinetoram group compared to the controls (Metabolic rate: *t*_5_ = 4.11; *p* = 0.008; water loss: *t*_5_ = 3.81; *p* = 0.011). As such, larvae fed on 0.01 mg/L Spinetoram diet, showed a CO_2_ release of 1.45 ± 0.03 μl/h/mg (*n* = 6) comparing to a control value of 0.64 ± 0.09 μl/h/mg (*n* = 5). The water loss of the Spinetoram and control individuals was 73.38 ± 32.42 μl/h/mg (*n* = 6) and 18.00 ± 3.61 μl/h/mg (*n* = 5), respectively. The CT_*max*_ value did not differ from the value determined with the control group — 46.91 ± 2.36°C (*n* = 11). We also recorded the metabolic rate per mg insect (Figure 3A and Table 1C) and the equivalent water loss values (Figure 3B and Table 1D) at different temperature points 25, 30, 35, 40, 45 ± 1°C. The most parsimonious model included toxin concentration, temperature with an interaction term. The stress phases in the two groups did not differ from each other (Figure 4A).

### Experiment 2: Azadirachtin Exposure

All larvae from the control group and the lowest Azadirachtin concentration survived the feeding trial, whereas larvae fed on higher concentrations suffered mortality. The survival rate of the larvae was significantly correlated with the toxin concentration in their food (*z*_36_ = −3.97; *p* < 0.001; Figure 1B). Larvae treated with 30 and 60 mg/L Azadirachtin died quickly after treatment started. We also observed that most larvae died during their ecdysal stage.

Larvae fed on toxin concentration of 30 and 60 mg/L Azadirachtin started gaining weight but could not maintain this throughout their juvenile stage, resulting in a reduction of weight over time. The control group was gaining weight every day despite a decrease in size due to the pre-pupal stage. All larvae of control group molted successfully to third-instar 3 days after the treatment started. At day 8, all caterpillars had molted to fourth-instar, and all reached final-instar at day 10. Caterpillars treated with 10 mg/L Azadirachtin had a reduced body mass gain comparing to control but did not lose weight as the larvae treated with higher concentrations (Figure 2B and Table 1D). Those caterpillars had molted to third-instar after 3 days. After 10 days, five of ten larvae had reached forth-instar and by the end of the trial seven had reached forth-instar while three remained in there third-instar.

In the higher Azadirachtin concentration of 30 mg/L, eight of ten caterpillars had molted to third-instar after 3 days of treatment. On day eight and ten of the remaining second-instars died and at day 13 only three third-instars remained. Finally, in the highest concentration of 60 mg/L Azadirachtin, three of ten caterpillars reached third-instar in 3 days. After 6 days two caterpillars died and another two, 2 days later. At day ten only 1 second- and one third-instar caterpillar remained, which both died by the end of the trail. The lowest AIC model for the body mass gain of larvae included the interaction between the concentration of Azadirachtin in diet and the duration of the experiment. For the growth (Figure 2C and Table 1F), the same model was chosen. The interaction term in the models was negative, indicating that the effect of toxin concentration became more pronounced over time.

Except for the highest toxin concentration tested, larvae form all groups completed at least one successful molt. According to the most parsimonious model (Table 1G) the timing of the successive molting stages was dependent on the larvae’s body mass as well as toxin concentration. Both factors were interrelated. As such, molting stages occurred later in larvae treated with Azadirachtin. Substituting log body mass with log length (Table 1H) resulted in an equivalently valid model.

Furthermore the dry food weight consumed by the control group (0.211 ± 0.07 g) was significant higher (*t*_15_ = −3.99; *p* = 0.001) than caterpillars fed on 10 mg/L Azadirachtin (0.09 ± 0.05 g).

### Metabolic Rate and Thermal Limit

Also in the Azadirachtin treatment, only larvae fed on the lowest concentration and the control caterpillars survived long enough for the metabolic rate measurements. *V̇*CO_2_ per mg body mass (control: 0.74 ± 0.11 μl/h/mg, *n* = 4; Azadirachtin; 1.72 ± 0.17 μl/h/mg, *n* = 7; *t*_8_ = 10.41; *p* < 0.001) as well as water loss per mg body mass (control: 23 ± 3.5 l/h/mg, *n* = 4; Azadirachtin: 97.89 ± 39.44, *n* = 7; *t*_6_ = 4.61; *p* = 0.003) was higher in Azadirachtin fed animals. The CT_*max*_ value was not significantly different between both groups and was on average 50.03 ± 2.46°C (*n* = 11). The most parsimonious model for mass-specific metabolic rate and the mass-specific water loss at certain temperature points included the concentration of toxin, temperature and interaction term. Mass-specific metabolic rate was significantly higher on different temperature points (Figure 3C and Table 1I). For the water loss/mg, caterpillars treated with Azadirachtin were not significantly different from the control group until 40°C (Figure 3D and Table 1J). The stress phases in response to the temperature were not significantly different in the tested groups (Figure 4B).

The control groups from both experiments (Azadirachtin and Spinetoram) were not significant different in there total mass-specific metabolic rate (*n* = 10; *t*_5_ = 1.27; *p* = 0.25), in their water loss (*n* = 10; *t*_7_ = 1.91; *p* = 0.10) and in the CT_*max*_ value (*n* = 10; *t*_7_ = 0.60; *p* = 0.57).

## Discussion

Here we tested which concentrations of Spinetoram and Azadirachtin were lethal to *H. punctigera* and which sub-lethal concentrations were effecting their growth and metabolic rate. Results showed that if applied early, 0.2–0.6 mg/L Spinetoram killed most larvae within 48 h, while 0.04–0.08 slowed down developmental duration or even prevented larvae from maturing and causing damage in laboratory conditions. When the closely related species, *H. armigera*, was exposed to a Spinosyn toxin (0.6 mg/Kg Spinosad in diet), it caused a mortality of 70% after 48 h of exposure, while lower concentrations reduced the growth and delayed molting stages; the lowest concentration of 0.04 mg/Kg resulted in a mortality of 10% and caused the female to lay only half of the number of eggs compared to a control (Wang et al., 2009). In another study, the lethal concentration required to kill 50% of the population (LC_50_) for *H. armigera* third instar is set to 0.08 mg/L (Dripps et al., 2008). Therefore, *H. punctigera* and *H. armigera* seemed to have the same susceptibility to Spinetoram. According to our and other studies Lepidoptera (LC_50_ 0.01 - 1.17 mg/L) are very sensitive to Spinetoram (Shimokawatoko et al., 2012). Thysanoptera are even more sensitive (LC_50_ 0.02 - 0.04 mg/L), but orders including Diptera (LC_50_ 23 mg/L) and Hemiptera (LC_50_ 47 mg/L) are more resilient (Shimokawatoko et al., 2012). These differences may be caused by the sensitivity of the receptors on their synapses, body mass and developmental stage (Shimokawatoko et al., 2012). For field use of Spinetoram, the manufacturer, Dow AgroSciences recommends 23 mg/L to control *Helicoverpa* genus on berry fruits, meaning the actual toxin concentration applied in a field is about 60 times higher than the death doses determined in the lab, this is also according to the surface application instead of mixing toxins into the food. Of course, laboratory results cannot be compared with field results, but laboratory test could be used as a first reference to field trail application.

Both insecticides could interfere with the growth of the larvae by disrupting the digestibility of the diet. At the end of the experiment, survival larvae fed on the lowest toxin concentration had eaten only 60% (Spinetoram) or 40% (Azadirachtin) of the amount the control group had consumed. Therefore, the smaller size of the caterpillars could be due to less food consumed, reduced digestibility or other growth regulation factors disturbed by the toxins. Also larvae treated with Azadirachtin were slower in taking up body mass than control group, ceased feeding and subsequently molting was incomplete. Other studies confirmed the influence of Neem oil on feeding behavior across different insect orders (Warthen, 1979; Jacobson, 1986; Schmutterer, 1990). Furthermore, the inhibition of the ecdysis seemed to be dose-dependent. Various studies confirm that Azadirachtin inhibits the molting process and causes death in different insect orders (Sieber and Rembold, 1983; Schmutterer, 1990). Only caterpillars treated with 10 mg/L Azadirachtin were able to molt to their fifth instar. In a study of De-Ling Ma et al. (2000) newly hatched and second instar caterpillars of the genus *Helicoverpa* were exposed to Azadirachtin mixed into an artificial diet as well as sprayed cotton plants. Even high concentrations of Azadirachtin, in the field, had a poor efficiency against *Helicoverpa* larvae, while when fed with 33 mg/L Azadirachtin in artificial diet resulted in death within a few days (Ma et al., 2000). In practice Neem oil or Azadirachtin is used for aphids, scale, thrips, whitefly, leafhoppers and weevils (Chandler et al., 2011).

To test the non-lethal effects of Spinetoram and Azadirachtin, we also assessed the metabolic rate of survived exposed *H. punctigera* caterpillars at different temperatures. Since *Helicoverpa* species are present in various states of Australia, they can be expected to be tolerant of a wide range of climatic conditions (Gregg et al., 2018). In all temperature, stages the insects treated with 0.01 mg/L Spinetoram or 10 mg/L Azadirachtin had a significant higher mass-specific *V̇CO*_2_ release than the control group. Insecticides could disturb metabolic function by causing higher energy consumption. The metabolic rate is usually higher in muscle tissue than fat tissue. Insects of the control group were developing faster than treated ones (53% lighter) and had more fat tissue due to the pre-pupal stage: this could also be a reason for a lower metabolic rate per mg tissue in the control group. Furthermore, caterpillars treated with insecticides released significant more water per mg insect, through their spiracles than the control group: this could be an indicator for a higher stress sensitivity in these insects. However, when taking into account the different phases of metabolic rate, equilibrium, ramping and pre-mortal plateau, we observed, that there was a correlation between temperature raising and *V̇*CO_2_ release, but there was no difference between the insects treated with insecticides and the ones without. Therefore, the toxins did influence the *V̇*CO_2_ per mg tissue, but did not cause a higher stress sensitivity to temperatures.

Insects have a tracheal system to transport gases, which can be changed dramatically through the spiracular opening (Harrison, 2009). Typically, the spiracles close only in short periods resulting in a continuously oxygen and carbon dioxide exchange (Harrison, 2009). If metabolic rates increased, because of higher temperature or perhaps stress level, the period between spiracular phases tended to decrease. Usually, this natural switching on and off of the discontinuous gas exchange has little effect on the water loss of the insect (Harrison, 2009). However, we observed that from a certain point of temperature increase, the caterpillars seemed to have lost control over the opening and closing of the spiracles. In contrast to the Spinetoram exposed insects, caterpillars treated with Azadirachtin lost significant more water over their spiracles above 40°C. This could be caused by partial melting of the cuticle lipid layer at 42°C, increasing permeability (Rourke, 2000). According to other studies from Lighton and Garrigan (1995) discontinuous gas exchange leads to water loss in caterpillars and ants, while in cockroaches (Noble-Nesbitt et al., 1995) and flies (Williams and Bradley, 1998; Williams et al., 1998) no significant correlation was found. For CT_*max*_ we found no differences between the treated insects and the control group. It is also unclear whether the larvae died because of overheating or desiccation.

To conclude, both toxins tested are sufficient to stop *Helicoverpa* species from feeding on artificial diets. However, the most effective doses that need to be applied in the field is usually much higher than in laboratory conditions. Furthermore, a very low concentration of toxin influences the fitness of the larvae. Poisoned insects consume more energy per mg tissue. The water loss rate of caterpillars treated with insecticides was higher. A non-lethal dose may prevent the insect from causing damage and therefore could lead to a reduction in the concentration of pesticide to be applied, particularly if the application could be formulated for direct ingestion. Further studies on developmental stages i.e., pupation and eclosion rate have to be made, to further understand the effects on non-lethal insecticide exposure.

## Data Availability Statement

All datasets generated for this study are included in the article/supplementary material.

## Author Contributions

AB carried out the experiments, performed the measurements, and wrote the manuscript with support from NA. NA helped to supervise the project. AB and NA conceived the original idea. Both authors discussed the results and commented on the manuscript.

## Conflict of Interest

The authors declare that the research was conducted in the absence of any commercial or financial relationships that could be construed as a potential conflict of interest.
